# The effect of weak acid anions on the selective catalytic wet air oxidation of aqueous ammonia to nitrogen

**DOI:** 10.1038/s41598-017-04207-5

**Published:** 2017-06-20

**Authors:** Zidan Wang, Sohaib Hameed, Yaoyao Wen, Nuowei Zhang, Hengjun Gai, Jinbao Zheng, Bing H. Chen

**Affiliations:** 1 Department of Chemical and Biochemical Engineering, College of Chemistry and Chemical Engineering, National Engineering Laboratory for Green Chemical Productions of Alcohols-Ethers-Esters, Xiamen University, Xiamen, 361005 PR China; 2 Qingdao University of Science and Technology, Shandong Qingdao, 266042 China

## Abstract

In this work, the effect of weak acid anions on the ammonia removal has been extensively studied for the process of selective catalytic wet air oxidation (CWAO) of ammonia to nitrogen. It is found that the presence of weak acid anions can effectively enhance the ammonia conversion and selectivity towards nitrogen. The combination between the weak acid anions and H^+^ to produce weak acid molecules is responsible for such enhancement. Firstly, the H^+^ consumption of weak acid anions can increase the NH_3_ concentration and thus the reactivity of ammonia oxidation, due to the shift to NH_3_ on the equilibrium of NH_4_^+^/NH_3_. Secondly, the competition combination with H^+^ between the weak acid anions and NO_2_^−^ can increase the concentration of NO_2_^−^ and thus boosts the disproportionation reaction between NH_4_^+^ and NO_2_^−^ to produce nitrogen.

## Introduction

Ammonia containing wastewater is one of the critical environmental issues, which causes serious eutrophication problems and is harmful to aquatic organisms. Several technologies, including biological nitrification, activated carbon adsorption and ion exchange processing, have been used to remove ammonia from wastewater before discharged. However, these methods are usually limited to be used in situation of low ammonia concentration and/or often inefficient^1–5^. Catalytic wet air oxidation (CWAO) of ammonia to N_2_ has been considered as one of the promising technology to deal with this issue, in which catalysts are used to selectively convert ammonia into N_2_^6–8^.

Supported transition metals and composite oxides have been utilized as the catalysts for decomposing ammonia from wastewater and shown excellent catalytic performance and potential under appropriate reaction conditions^9–19^. Meanwhile, the reaction behavior and mechanism of CWAO of ammonia to N_2_ has also been investigated on the purpose of helping to understand the reaction, so as to guide the catalyst development. In order to increase ammonia removal efficiency, strong alkaline were usually added into the feed solution, since ammonia mainly exists as NH_3_ in alkaline region and most of studies convinced that NH_3_ is much more reactive than NH_4_^+^ on the CWAO of ammonia. Qin *et al* compared the reactivity of NH_4_^+^ and NH_3_ by adjusting the initial pH in the range from 3 to 13^20^. It was found that ammonia oxidation occurred at pH above 10, in which ammonia mainly exists as NH_3_ rather than NH_4_^+^. They thought the N_2_ formation was from the catalytic surface reaction, and the NO_3_^−^ was from the further oxidation of NO_2_^−^. Based on their experiments and investigations, following mechanism was proposed:

1 $${{\rm{O}}}_{2}+2\ast \to 2{{\rm{O}}}^{\ast }$$

2 $${{\rm{NH}}}_{3}+{{\rm{O}}}^{\ast }\to {{\rm{NH}}}^{\ast }+{{\rm{H}}}_{2}{\rm{O}}$$

3 $${{\rm{NH}}}^{\ast }+{{\rm{O}}}^{\ast }\leftrightarrow {{\rm{HNO}}}^{\ast }+\ast $$

4 $${{\rm{NH}}}^{\ast }+{{\rm{HNO}}}^{\ast }\to {{\rm{N}}}_{2}+{{\rm{H}}}_{2}{\rm{O}}+2$$

5 $${{\rm{HNO}}}^{\ast }+{{\rm{O}}}^{\ast }\leftrightarrow {{\rm{HNO}}}_{2}^{\ast }+\ast $$

6 $${{\rm{HNO}}}_{2}^{\ast }\leftrightarrow {{\rm{NO}}}_{2}^{-}+{{\rm{H}}}^{+}+\ast $$

7 $${{\rm{NO}}}_{2}^{-}+{{\rm{O}}}^{\ast }\leftrightarrow {{\rm{NO}}}_{3}^{-}+\ast $$

where, *indicates the catalytic active site at vacant state.

Lee *et al* ^21, 22^ also convinced that the reaction of CWAO of ammonia to N_2_ proceeded rapidly in strong alkaline region such as pH>10. However, they thought that N_2_ was solely produced from the homogeneous reaction between NO_2_^−^ and NH_4_^+^ and the formation of HNO_2_ was responsible for the pH decreasing based on the fact that the pH of the solution was decreased from 12 to 2 after reaction. Accordingly, the following reaction pathways partly overlapped the mechanism by Qin *et al* were proposed:

1 $${{\rm{O}}}_{2}+2\ast \to 2{{\rm{O}}}^{\ast }$$

2 $${{\rm{NH}}}_{3}+{{\rm{O}}}^{\ast }\to {{\rm{NH}}}^{\ast }+{{\rm{H}}}_{2}{\rm{O}}$$

3 $${{\rm{NH}}}^{\ast }+{{\rm{O}}}^{\ast }\leftrightarrow {{\rm{HNO}}}^{\ast }+\ast $$

5 $${{\rm{HNO}}}^{\ast }+{{\rm{O}}}^{\ast }\to {{\rm{HNO}}}_{2}+2\ast $$

8 $${{\rm{HNO}}}_{2}\leftrightarrow {{\rm{NO}}}_{2}^{-}+{{\rm{H}}}^{+}$$

9 $${{\rm{NO}}}_{2}^{-}+{{\rm{NH}}}_{4}^{+}\to {{\rm{N}}}_{2}+2{{\rm{H}}}_{2}$$

7 $${{\rm{NO}}}_{2}^{-}+{{\rm{O}}}^{\ast }\leftrightarrow {{\rm{NO}}}_{3}^{-}+\ast $$

Lousteau *et al* reported that part of nitrites was reduced to molecular nitrogen via a retro-disproportionation reaction between the ammonium and nitrites ions (NH_4_^+^+ NO_2_^−^ = N_2_+ 2H_2_O) while part of them was oxidized into NO_3_^−^ by oxygen. The lower pH (towards acidic condition), the more NO_3_^−^ was produced. They also found that Pd/TiO_2_ catalyst can shift the HNO_2_/NO_2_^−^ equilibrium toward the basic form (NO_2_^−^) because of high pH (>8), which inhibited one possible evolution route toward the formation of nitrates and in turn “promoted” the retro-disproportionation reaction toward molecular nitrogen formation^12^.

There are two key equilibriums in the process for CWAO of ammonia to N_2_:

10 $${{{\rm{NH}}}_{4}}^{+}\leftrightarrow {{\rm{NH}}}_{3}+{{\rm{H}}}^{+}$$

11 $${{\rm{HNO}}}_{2}\leftrightarrow {{{\rm{NO}}}_{2}}^{-}+{{\rm{H}}}^{+}$$

The equilibrium 10 is related to the catalytic oxidation reaction and the equilibrium 11 to the retro-disproportionation reaction between NH_4_^+^ and NO_2_^−^. It is reasonable to deduce that the addition of the substance that can consume H^+^ in the feed solution will shift the both equilibrium to right. The increasing ammonia concentration will facilitate the oxidation of ammonia and the increasing NO_2_^−^ concentration will favor the formation of N_2_ and inhibit the NO_3_^−^. However, as far as we know, no such work can be found in literature.

In this paper, the weak acid anions were introduced into the feed solution to enhance the removal efficiency of ammonia and the selectivity to N_2_. As expected, the addition of weak acid anion can combine with H^+^ and adjust the concentration of NH_3_ and NO_2_^−^ during the process of CWAO of ammonia, which effectively improve the catalytic performance.

## Experimental Section

### Catalyst preparation

Three different kinds of catalysts, Ru-Cu/C, Pt/TiO_2_ and Ru/TiO_2_, were synthesized to compare the influence of weak acid anions on the catalytic wet air oxidation of ammonia to nitrogen. H_2_PtCl_6_·6H_2_O aqueous solution and RuCl_3_·3H_2_O aqueous solution were used as noble metal precursors, where the concentration were accurately determined by inductively coupled plasma-optical emission spectroscopy (ICP-OES). CuCl_2_.2H_2_O was also used as active metal precursor.

Pt (3 wt%) supported on TiO_2_ (Sinopharm Chemical Reagent Co., Ltd. surface area: 10 m^2^/g) were prepared via incipient wetness impregnation method^10^. Ru (3 wt%) supported on TiO_2_ (Degussa P-25 Surface area: 50 m^2^/g) and bimetallic catalyst Ru-Cu/C were synthesized by the reduction method described in our previous work^23^. The amount of precursor was adjusted to obtain 1.5% metal loading of Ru and 1.5% metal loading of Cu for the bimetallic catalyst. All the obtained solids were dried at 60 °C in the oven for one night and finally Ru catalysts were calcined at 250 °C and Pt catalyst reduced at 300 °C in flowing hydrogen (4.8 Lh^−1^) for 4 h in the furnace.

### Catalyst characterization

The specific surface area of the catalyst was determined by N_2_ adsorption (the BET method) using a Micromeritics Tristar 3000 instrument. The chemical composition of the sample was measured by inductively coupled plasma atomic emission spectroscopy (ICP-AES). X-ray powder diffraction (XRD) patterns were collected over a Panalytical X’pert Pro diffractometer operated at 40 kV and 30 mV, using Cu *Kα* radiation. The images of TEM were obtained on a Tecnai F30 electron microscope (Phillips Analytical) operated with an acceleration voltage of 300 kV.

### Apparatus and reaction

The feed solutions for the evaluation of different acid anions were prepared as following: (1) Add 0.55 mL of ammonia solution (mass percentage = 25%) into deionized water to prepare a 100 mL solution containing 1000 ppm ammonia. (2) Take 10 mL solution of (1) and then add the corresponding acid solution drop by drop to adjust the pH to around 8.5. Record the volume of the used acid solution (V). (3) Mix 0.55 mL of ammonia solution (25%) with 10V acid solution, and then deionized water was added into the mixture to 100 mL. The feed solutions for the evaluation of different pH were prepared by dissolving the desired NH_4_Cl or CH_3_COONH_4_ into deionized water and then adding 2M NaOH to adjust pH.

The catalytic performance of CWAO of ammonia to N_2_ were tested in a 100 mL stainless steel autoclave reactor coated with Teflon liner 2 mm thick inside to avoid corrosion of the reactor wall and a magnetic spin bar was used for stirring. In a typical run, 0.6 g of catalyst and 60 mL reaction solution containing 1000 ppm ammonia was added in the reactor. After adjusting the initial pH to a desired value, the reactor was purged with high purity N_2_ five times in order to remove the trace of O_2_. Then, the mixture was heated to the desired temperature under stirring at a rate of 1000 rpm. When the temperature was reached to desired value, 2 MPa air was immediately introduced into the reactor. This moment was considered as zero (starting) time for reaction. Liquid samples were extracted from the reactor at regular intervals of time and filtered by a membrane filter (pore size: 1 μm). After the collected liquid samples were cooled to the ambient temperature, the pH and concentrations of ammonia, NO_3_^−^ and NO_2_^−^ were analyzed.

For the reaction behavior between NH_4_^+^ and NO_2_^−^, 10 mL solution containing the same concentrations of NH_4_^+^ and NO_2_^−^ (500 ppm) was introduced into the reactor. The initial pH of the feed was adjusted to 6.2. Then, the air was introduced and the temperature was increased to 150 °C with a stirring rate of 1000 rpm. 3 hours later, the reactor was separated from the heater and cooled to room temperature. Finally, the liquid sample was treated and analyzed.

### Product Analysis

The liquid samples were analyzed by Napierian reagent calorimetric method (GBZ5000587) using UV detector at 405 nm to measure the ammonia-nitrogen. The further oxidation products of ammonia (NO_2_^−^ and NO_3_^−^) in the liquid phase were measured by HPLC (High Performance Liquid Chromatography) and LC/MS (liquid chromatography/mass spectrometry). The gaseous products were analyzed by a GC equipped with two columns (Porapack T and Molsieve 5A).

As the detected nitrogen containing compounds were only N_2_, NH_3_, NH_4_^+^, NO_3_^−^ and NO_2_^−^, the conversion of ammonia (${{\rm{x}}}_{{{\rm{NH}}}_{3}}$) and selectivity of nitrogen (${{\rm{S}}}_{{{\rm{N}}}_{2}}$) was calculated by the following formulas:

$${{\rm{x}}}_{{{\rm{NH}}}_{3}}( \% )=\frac{{C}_{N{H}_{3}}^{0}-{C}_{N{H}_{3}}\,}{{C}_{N{H}_{3}}^{0}}\times 100 \% $$

$${{\rm{S}}}_{{{\rm{N}}}_{2}}( \% )=(1-\frac{{C}_{N{O}_{3}^{-}}+{C}_{N{O}_{2}^{-}}\,}{{C}_{N{H}_{3}}^{0}-{C}_{N{H}_{3}}})\times 100 \% \,$$

where C^0^ is the initial concentration of the substrate in the solution, C is its current concentration and the subscripts denote the individual compounds or ions.

The pH values of reaction solution before and after oxidation were measured using a pH meter (Radiometer Analytical PHM240).

Total organic carbon (TOC) content in the liquid samples was measured by TOC-VCSH analyzer which employs high temperature combustion oxidation, coupled with non-dispersive infrared detection technology.

## Results and Discussion

### Characterization of catalyst

**Figure 1:**
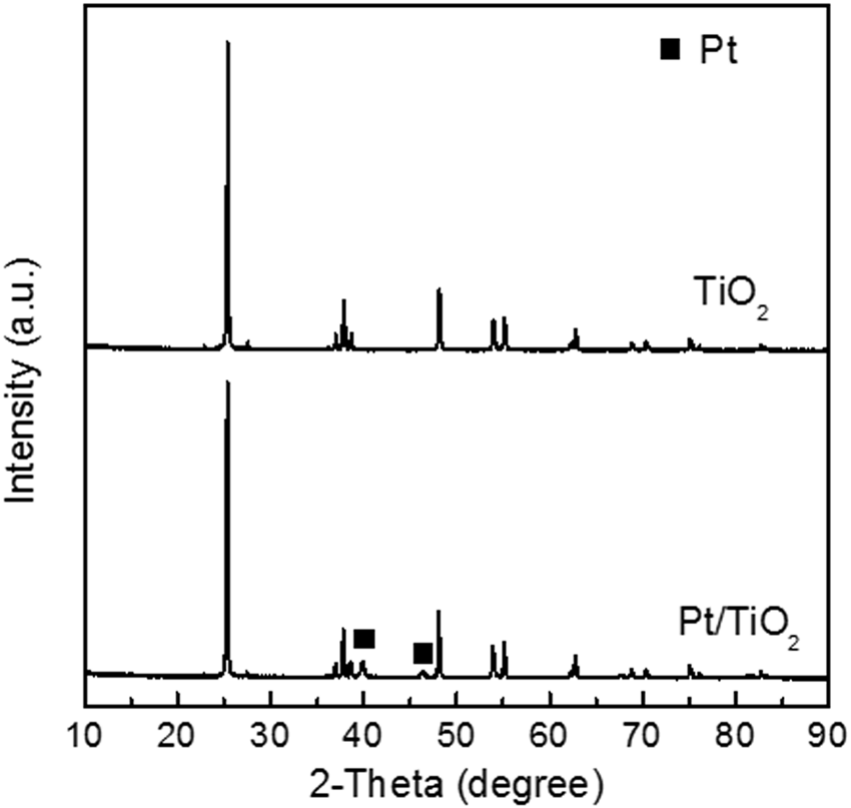
The XRD patterns of the TiO_2_ and Pt/TiO_2_.

**Figure 2:**
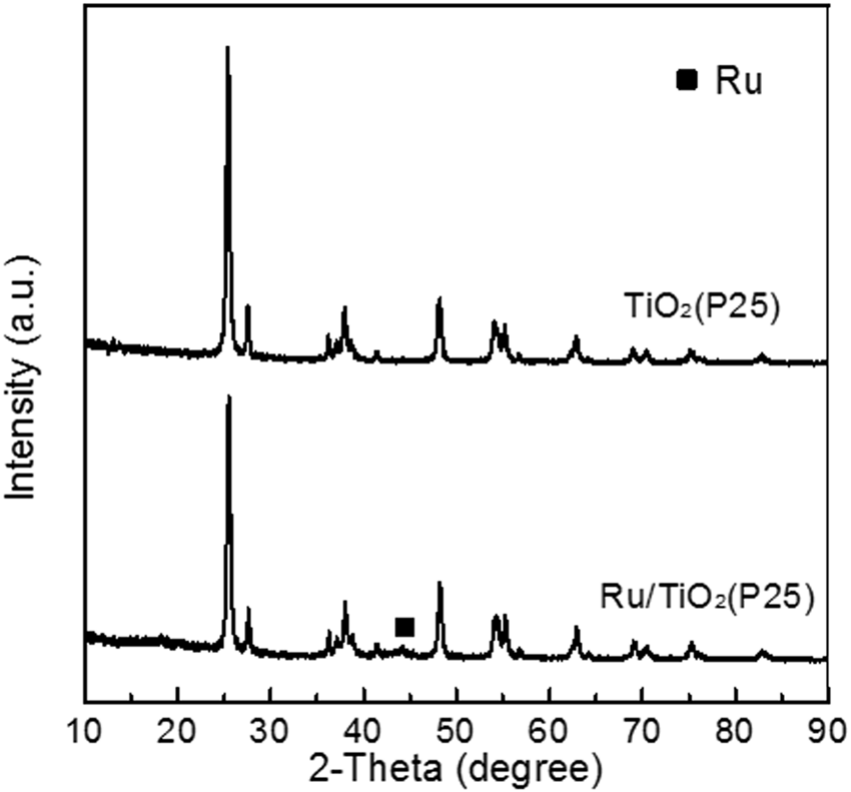
The XRD patterns of the TiO_2_ and Ru/TiO_2_.

**Figure 3:**
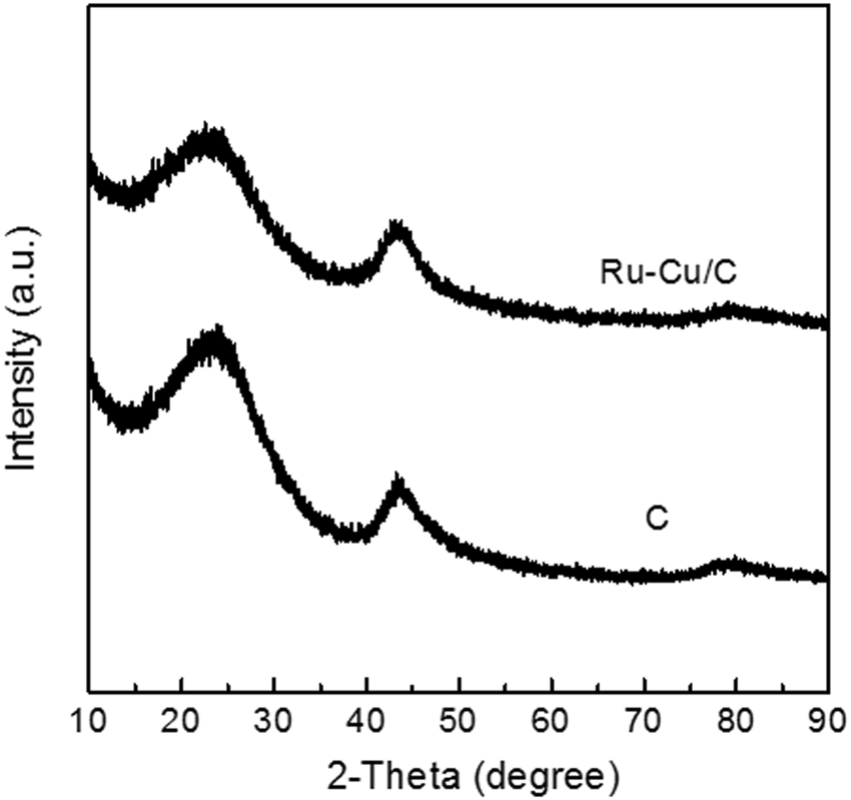
The XRD patterns of the C and Ru-Cu/C.

**Figure 4:**
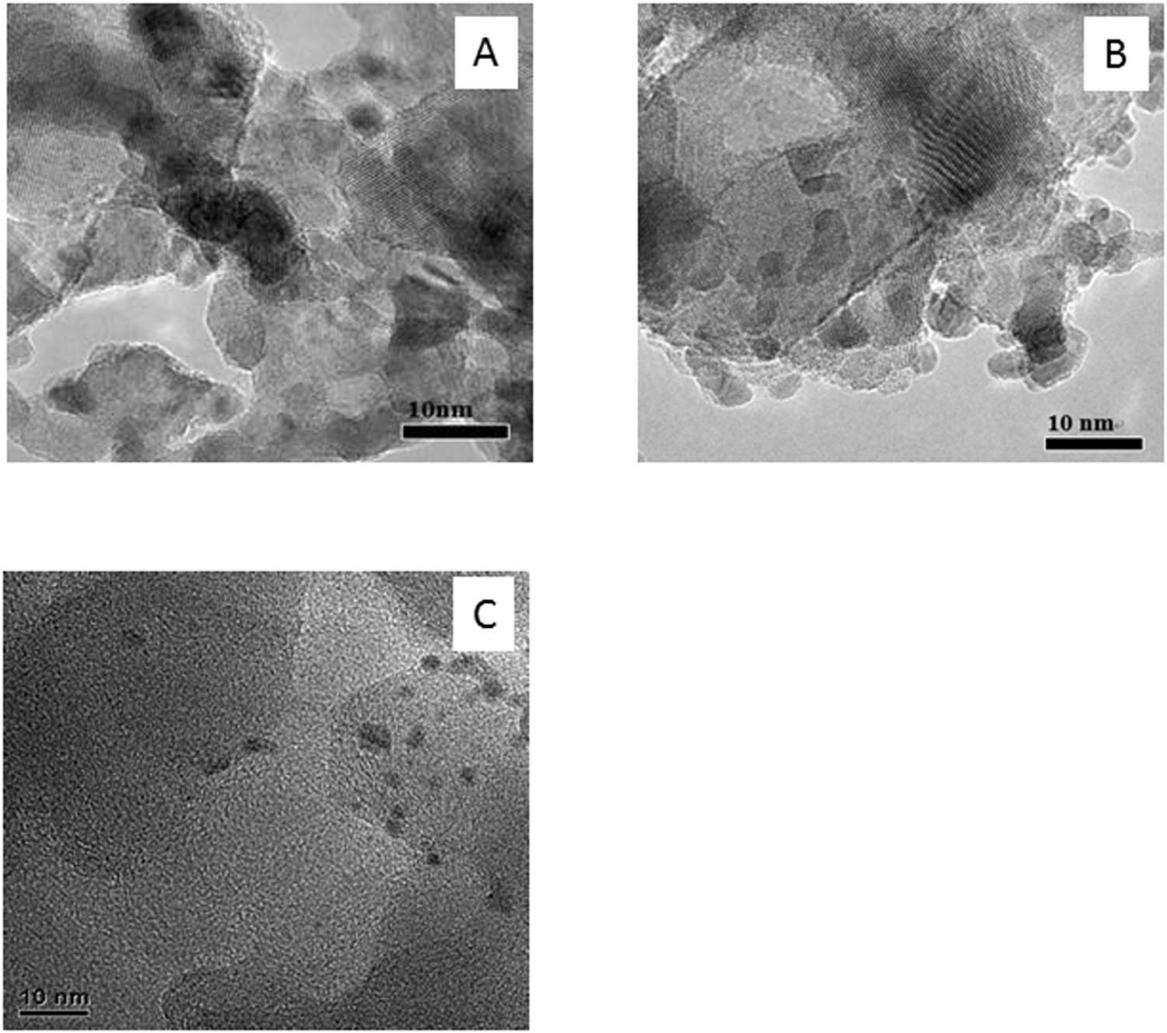
TEM images of Pt/TiO_2_ (**A**), Ru/TiO_2_ (**B**) and Ru-Cu/C (**C**).

The Pt/TiO_2_, Ru/TiO_2_ and Ru-Cu/C catalysts were characterized by ICP, N_2_ adsorption, XRD and TEM before catalytic reaction. As shown in Table 1, the loadings of Pt and Ru over Pt/TiO_2_ and Ru/TiO_2_ catalysts were 2.86 and 2.89%, respectively. For Ru-Cu/C catalyst, the loadings of Ru and Cu were 1.45 and 1.43%. These results indicated that most of active metals were successfully loaded on support as the measured loadings are quite close to the feed. The XRD patters of Pt/TiO_2_, Ru/TiO_2_ and Ru-Cu/C catalysts are shown in Figs 1, 2 and 3. For the sake of comparison, the corresponding supports are also shown. The similar XRD patterns of catalysts with supports indicated that the main structure of the supports were retained after the introduction of active metals. Signals for Pt and Ru metals were detected over Pt/TiO_2_ and Ru/TiO_2_ suggesting that the Pt or Ru species was mainly present in the form of metallic state and was aggregated to some extent, as shown in Fig. 4. No Ru or Cu species was detected over Ru-Cu/C catalyst, which is consistent with our previous study that both Ru and Cu mainly existed in the form of metallic state and was well-dispersed over carbon support^23^. Compared with Ru/TiO_2_, Pt/TiO_2_ exhibited more sharp XRD peaks of TiO_2_ suggesting that the support for Pt/TiO_2_ possessed much better crystal structure with the lowest the specific surface area (as shown in Table 1).

**Table 1:** The chemical composition and specific surface area of t Pt/TiO_2_, Ru/TiO_2_ and Ru-Cu/C catalysts.

Catalyst	Metal loading (%)	S_BET_ (cm^2^/g)
Pt/TiO_2_	Pt: 2.86	9.8
Ru/TiO_2_	Ru: 2.89	55.8
Ru-Cu/C	Ru: 1.45, Cu: 1.43	1389.2

### The effect of different acid anions on ammonia conversion

The effects of different acid anions on the ammonia conversion were studied by using ammonium phosphate, ammonium acetate, ammonium sulfate, ammonium chloride and ammonium nitrate as ammonia resource, respectively. The feed solutions were prepared by the procedure described in Experiment section. The experiments were performed at 150 °C and the results are shown in Table 2. It can be seen that the acid anion showed strong effects on the ammonia conversion. Ammonia conversion reached to a value higher than 30% when the feed solution contained weak acid anion. However, for the feed solution containing strong acid anion, only 10% ammonia was converted. To explore the effects of the weak acid anions on the catalytic activity, the reaction behavior of CWAO of ammonium were comparatively investigated by using ammonium acetate and ammonium chloride aqueous solutions in the following study.

**Table 2:** Effect of different anions on the ammonia conversion for CWAO of ammonia to N_2_ over Ru-Cu/C catalyst.

Solution	pH before reaction	Ammonia conversion/%
(NH_4_)_3_PO_4_	8.6	30.8
CH_3_COONH_4_	8.5	34.2
(NH_4_)_2_SO_4_	8.9	9.5
NH_4_Cl	8.4	10.6
NH_4_NO_3_	8.5	8.2

Conditions: [NH_4_^+^]_0_ = 1000 ppm, T = 150 ^o^C, Air pressure = 2 MPa, reaction time = 3 h.

### The effect of pH on ammonia conversion

**Figure 5:**
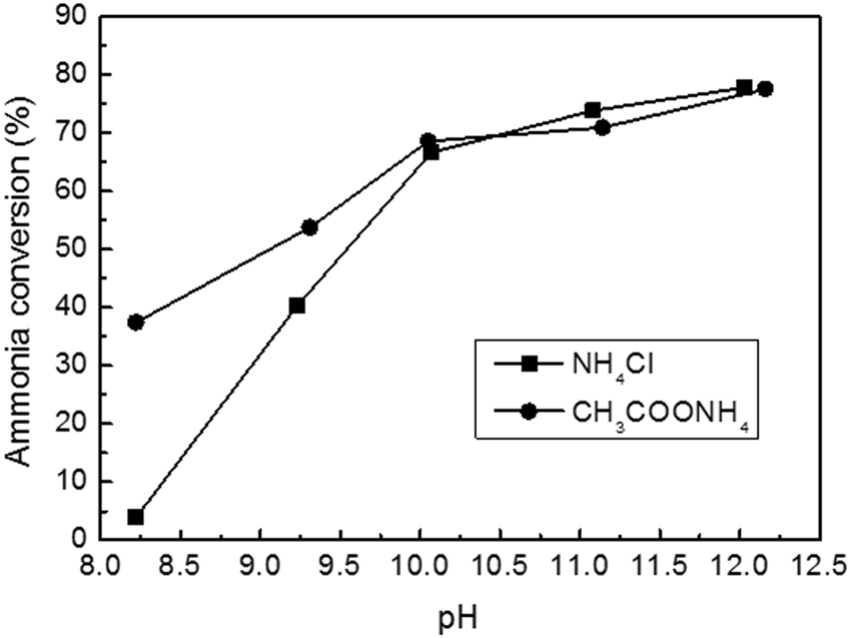
Effect of initial pH on ammonia conversion of NH_4_Cl and CH_3_COONH_4_ solutions over Ru-Cu/C catalyst. Conditions: [NH_4_^+^]_0_ = 1000 ppm, T = 150 ^o^C, Air pressure = 2 MPa, reaction time = 3 h.

The effects of pH on ammonia conversion were studied and the results are shown in Fig. 5. For convenience, the ammonia in ammonium acetate solution was denoted as ammonia-A and that in ammonium chloride solution as ammonia-C. As reported in literature, the initial pH strongly affected the ammonia conversion. The higher pH, the more ammonia was converted. This further convinces that NH_3_ is much more active than NH_4_^+^ in the CWAO of ammonia. It can also be seen that the presence of acetate can enhance the reactivity of ammonia, however, such enhancement was vulnerable to pH change. At pH 8.3, ammonia-A showed a conversion of 37.4% while only 3.9% of ammonia-C was converted. With pH increasing, the gap between conversions of ammonia-A and ammonia-C was narrowed. When pH was higher than 10, no obvious difference between ammonia-A and ammonia-C conversions was observed.

Since NH_3_ is more reactive than NH_4_^+^, one possible reason for the enhancement of ammonia conversion of ammonia-A at low pH was that CH_3_COO^−^ can increase the NH_3_ concentration via combining with H^+^. There are 3 reactions responsible for the H^+^ production during the process for CWAO of ammonia: (1) the dissociation of NH_4_^+^ (NH_4_^+^↔NH_3_+H^+^); (2) the dissociation of H_2_O (H_2_O↔H^+^+OH^−^); (3) the dissociation of HNO_2_ (HNO_2_↔H^+^+ NO_2_^−^). The combination between CH_3_COO^−^ and H^+^ can shift the 3 equilibrium to the right. For reaction (1), the shifting to right can increase the NH_3_ concentration. For the reaction (2), the shifting to right increases the OH^−^ concentration, which also increases the NH_3_ concentration due to the reaction between the OH^−^ and NH_4_^+^ to produce NH_3_ and H_2_O. For the reaction (3), the H^+^ consumption prevents the pH declining and thus increases the NH_3_ concentration, which will be proved later in this work.

### The effect of temperature on ammonia conversion and N_2_ selectivity

**Figure 6:**
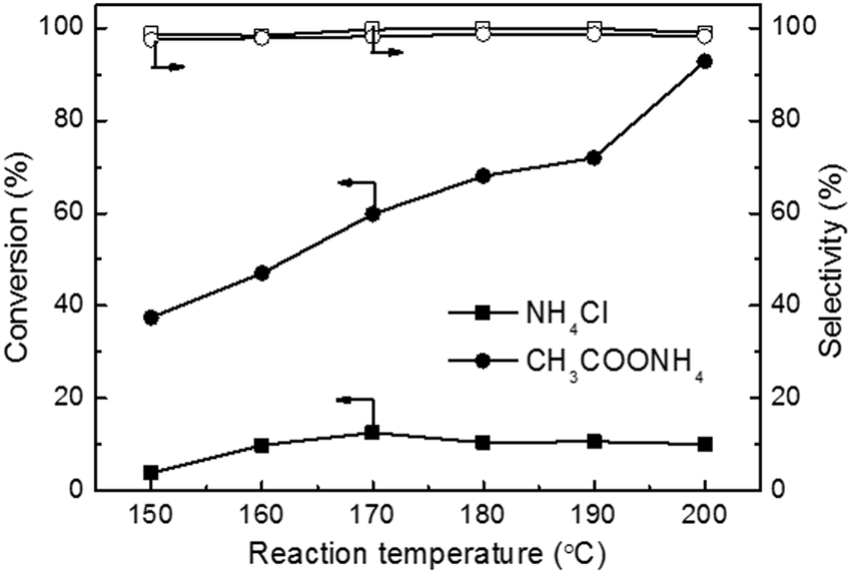
Effect of temperature on ammonia conversion and N_2_ selectivity of NH_4_Cl and CH_3_COONH_4_ solutions over Ru-Cu/C catalyst. Conditions: [NH_4_^+^]_0_ = 1000 ppm, pH = 8.2, Air pressure = 2 MPa, reaction time = 3 h.

Figure 6 shows the effects of reaction temperature on the ammonia conversion and N_2_ selectivity of ammonia-A and ammonia-C. When the initial pH was 8.2, the conversion of ammonia-A was always higher than that of ammonia-C and continuously increased with temperature ascending. When temperature was increased from 150 °C to 200 °C, the conversion was increased from 37.4% to 92.8%. In contrast, ammonia-C kept very low ammonia conversions (<11%) in the whole range of temperature. Temperature had no observable effect on the ammonia conversion for ammonia-C, which is consistent with literatures. M. Bernardi et al reported that the NH^4+^ ions were very reactive under moderately acid pH and catalytic wet air oxidation of ammonia can work well at near neutral pH conditions. A high NH_3_ conversion was obtained when the reaction was carried out in ammonium acetate aqueous solutions (pH = 6.8)^10^. Another study got more than 90% ammonia decomposition when a phosphate buffered solution (pH  = 6.8) was used in order to stabilize the acidic form^24^. However, when ammonium chloride was used as feed solutions, a high ammonia conversion was obtained only until the pH was reached up to 10^20–22^.

Two factors affect the reaction rate of ammonia oxidation reaction: the NH_3_ concentration and reaction temperature. The higher NH_3_ concentration, the higher reaction rate can be obtained. High temperature favors the ammonia oxidation and enables catalyst to reach a high ammonia conversion. Although the two systems (ammonia-A and ammonia-C) exhibited a similar initial pH, the presence of CH_3_COO^−^ can increase the NH_3_ concentration. Therefore, increasing temperature can increase the ammonia conversion of ammonia-A to a great extent, probably due to the available NH_3_. For ammonia-C, NH_4_^+^ is more predominant than NH_3_. Ammonia-C remained a very low ammonia conversion in the whole range of 150 to 200 °C due to the lack of NH_3_, since NH_4_^+^ can be hardly oxidized in CWAO process at temperatures lower than 200 °C.

### The effect of temperature on TOC conversion

**Figure 7:**
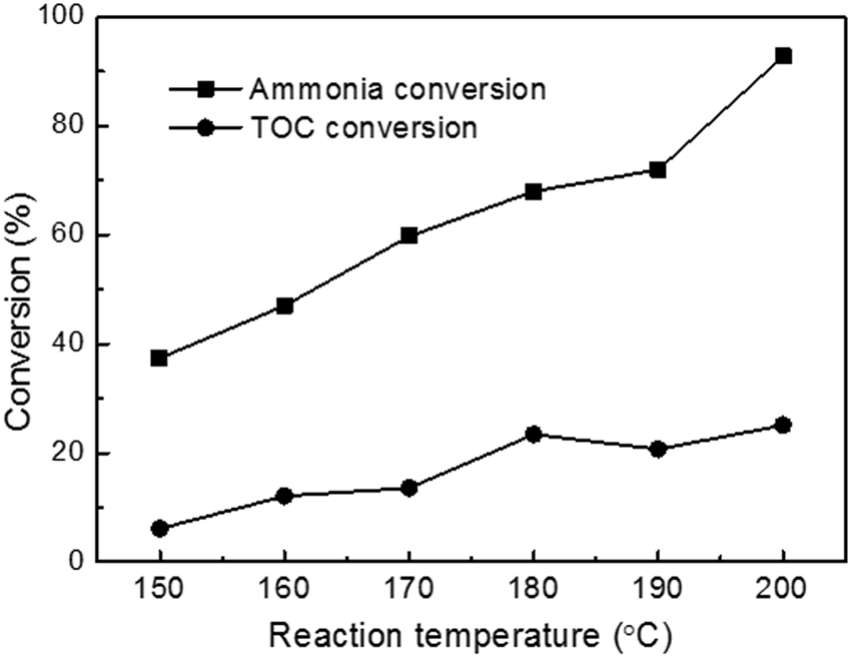
Effect of temperature on ammonia and TOC conversion of CH_3_COONH_4_ solution over Ru-Cu/C catalyst. Conditions: [NH_4_^+^]_0_ = 1000 ppm, pH = 8.2, Air pressure = 2 MPa, reaction time = 3 h.

During the CWAO process, acetate anions can be oxidized to CO_2_. The TOC conversion of ammonia-A as a function of reaction temperature is shown in Fig. 7. It can be seen that TOC conversion kept at a very low level, although it increased with increase of temperature. Even at 200 °C, the TOC conversion was only 25.1%. This result indicated that most of acetate anions were not oxidized and remained in the solution, which was in agreement with the reported data in the literature that acetic acids was refractory to further oxidation. No organic component but CH_3_COO^−^ was detected in the liquid phase after reaction, indicating that the final product of CH_3_COO^−^ oxidation was carbonate ions or carbon dioxide.

### Catalytic performance of Pt/TiO_2_ and Ru/TiO_2_ catalyst

**Figure 8:**
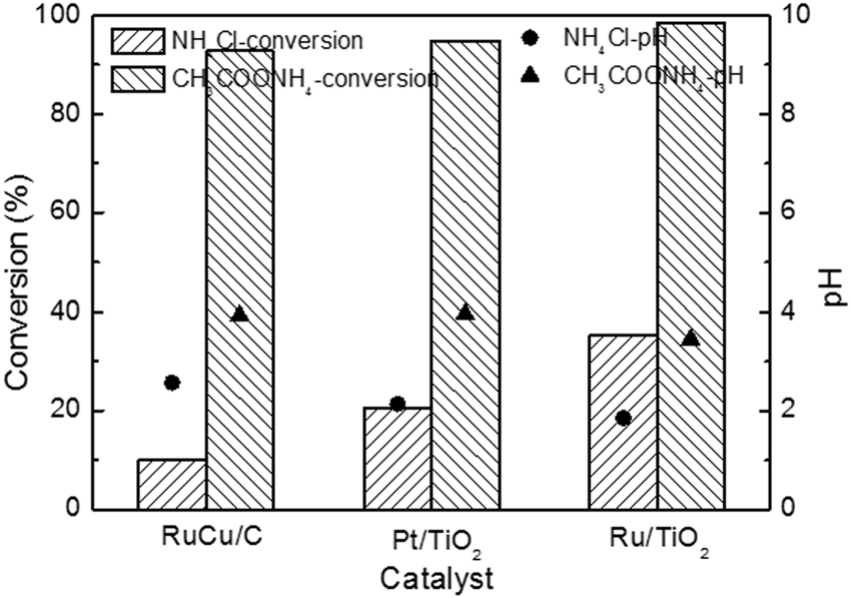
Ammonia conversion and pH after reaction of NH_4_Cl and CH_3_COONH_4_ solutions over Ru-Cu/C, Pt/TiO_2_ and Ru/TiO_2_ catalysts. Conditions: [NH_4_^+^]_0_ = 1000 ppm, pH = 8.2, T = 200 ^o^C, oxygen pressure = 2 MPa, reaction time = 3 h.

The positive effect of acetate anions on the ammonia conversion was further investigated over another two catalysts, Pt/TiO_2_ and Ru/TiO_2_, and the results are shown in Fig. 8. For comparison, Ru-Cu/C catalyst was also included. Similar enhancements for ammonia conversion were observed over the both catalysts, although Pt/TiO_2_, Ru/TiO_2_ and Ru-Cu/C catalysts exhibited different specific surface area and active metal particle size. More than 90% ammonia in ammonia-A can be converted over Pt/TiO_2_ and Ru/TiO_2_. However, just around 20% and 40% of ammonia was oxidized over Pt/TiO_2_ and Ru/TiO_2_ respectively, when the feed was switched to ammonia-C aqueous solutions. The pH after reaction is also presented in Fig. 8. It can be seen that, whatever catalyst, the feed solution of ammonia-A always had higher pH after reaction than that of ammonia-C, even although the initial pH were 8.2 for both cases. The possible reason for the higher final pH of ammonia-A is that CH_3_COO^−^ combined with H^+^ to produce CH_3_COOH and prevented the pH decreasing to some extent. Noted that the final pH of ammonia-A was lower than 5.0. Therefore, it is reasonable to think that the final oxidative species of CH_3_COO^−^ should be carbon dioxide, since carbonate ions will be converted into CO_2_ under such acidic condition.

### Evolutions of ammonia conversion and pH

**Figure 9:**
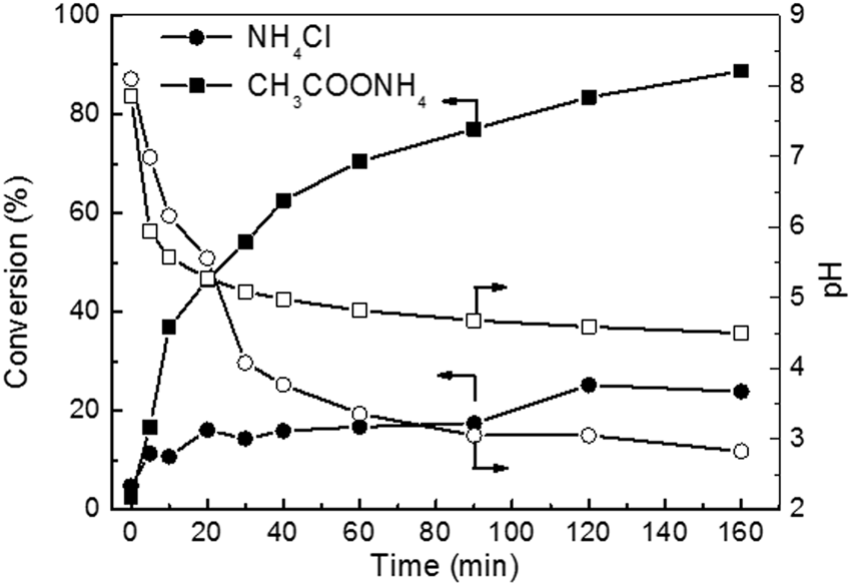
Evolutions of ammonia conversion and pH of NH_4_Cl and CH_3_COONH_4_ solutions over Ru-Cu/C catalyst. Conditions: [NH_4_^+^]_0_ = 1000 ppm, initial pH = 8.2, T = 200 ^o^C, Air pressure = 2 MPa.

**Figure 10:**
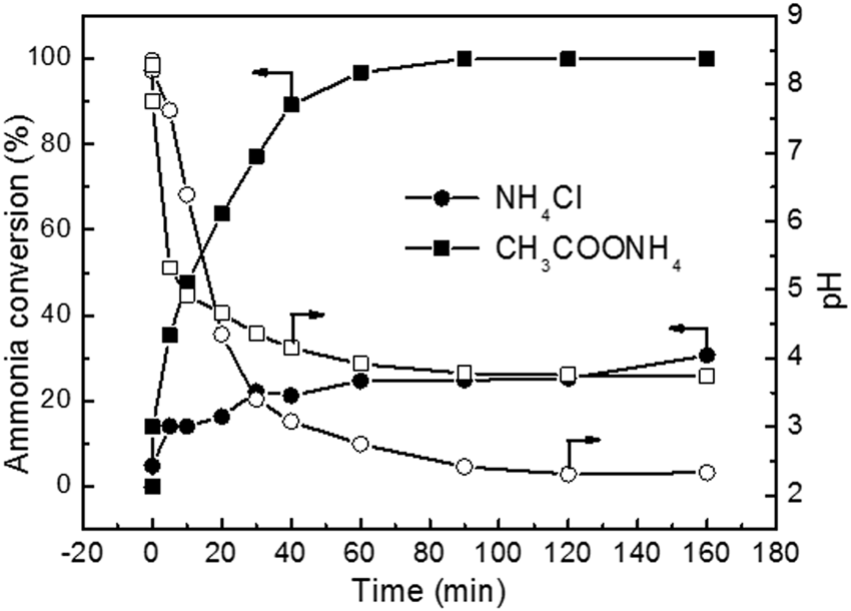
Evolutions of ammonia conversion and pH of NH_4_Cl and CH_3_COONH_4_ solutions over Pt/TiO_2_ catalyst. Conditions: [NH_4_^+^]_0_ = 1000 ppm, initial pH = 8.2, T = 200 ^o^C, Air pressure = 2 MPa.

**Figure 11:**
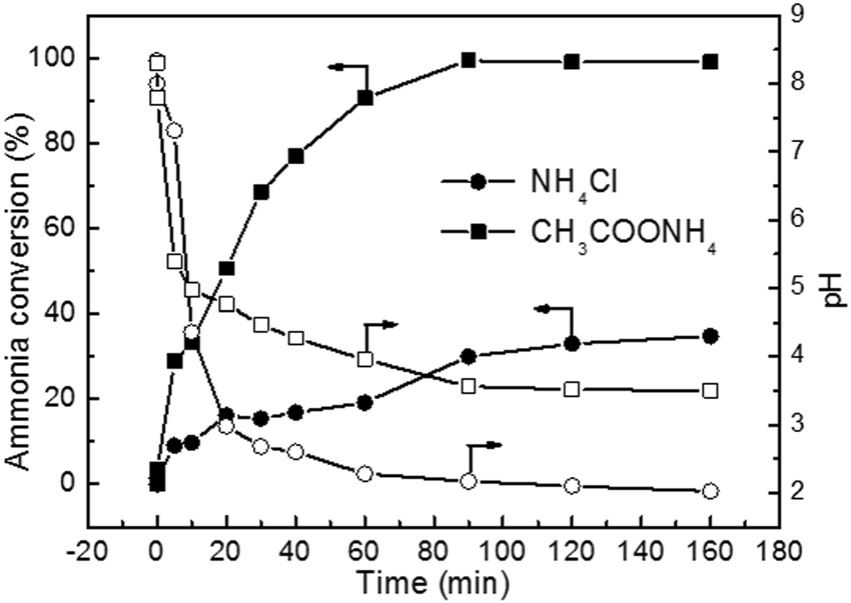
Evolutions of ammonia conversion and pH of NH_4_Cl and CH_3_COONH_4_ solutions over Pt/TiO_2_ catalyst. Conditions: [NH_4_^+^]_0_ = 1000 ppm, initial pH = 8.2, T = 200 ^o^C, Air pressure = 2 MPa.

**Figure 12:**
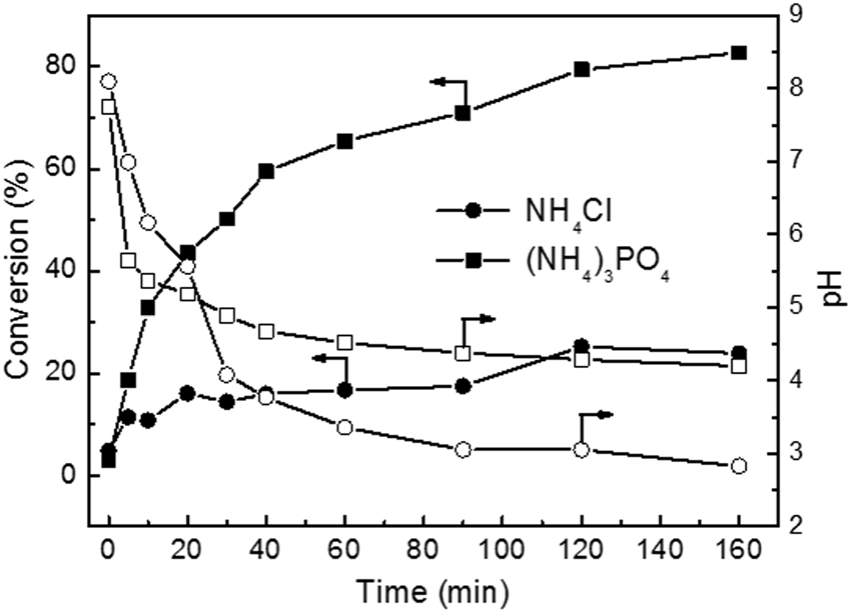
Evolutions of ammonia conversion and pH of NH_4_Cl and (NH_4_)_3_ PO_4_ solutions over Ru-Cu/C catalyst. Conditions: [NH_4_^+^]_0_ = 1000 ppm, initial pH = 8.2, T = 200 ^o^C, Air pressure = 2 MPa.

Figure 9 shows the evolutions of ammonia conversion and pH as a function of time over RuCu/C catalyst. It exhibited much higher ammonia conversions for ammonia-A than the ammonia-C. The ammonia conversion continuously increased with the reaction proceeding and reached a value of 88.7% after 160 minute. For ammonia-C, the ammonia conversion remained very low in the whole process, after 20 min reaction, the ammonia conversion was only 16.1% and the final conversion was 23.9%. In contrast with conversion, the pH decreased as the reaction proceeded in both solutions. In the first 20 minute, the pH decreased more rapidly for ammonia-A and thus was lower than that for ammonia-C. However, after 20 minute, a higher pH was observed for ammonia-A than that for ammonia-C. Generally, the pH decreasing during the CWAO of ammonia is related to the over-oxidation of ammonia to nitrogen oxide rather than nitrogen. At 200 °C, the ammonia is favorably oxidized to nitrous acid. Nitrous acid dissociates into a NO_2_^−^ ion and an H^+^ and thus lowers the pH of the solution. At the beginning of the reaction, more ammonia of ammonia-A was oxidized to nitrite (much higher ammonia conversions) and thus the pH drops down quicker than that for ammonia-C. After 30 min, a higher pH is exhibited for ammonia-A than ammonia-C, although conversion of ammonia in ammonia-A further increased and was much higher than in ammonia-C. These can be explained below. There are two reactions for ammonia consumption: ammonia oxidation and the disproportionation reaction between NH_4_^+^ and NO_2_^−^. At acidic pH, ammonia is mainly present in the form of NH_4_^+^ and the disproportionation reaction between NH_4_^+^ and NO_2_^−^ was responsible for the ammonia consumption. After 30 min, the pH in ammonia-A was lower than 5.1. Therefore, the conversion of ammonia-A kept increasing while the evolution of pH exhibited a very flat pattern, implying the disproportionation reaction is dominant in this stage when weak acid is presented. For ammonia-C, the flat pattern of pH evolution was contributed to the fact that only very little ammonia was oxidized after 30 min and nearly no disproportionation reaction happened. Very similar results can also be obtained over Pt/TiO_2_ and Ru/TiO_2_, as shown in Figs 10 and 11. The evolutions of ammonia conversion and pH were also studied in a phosphate buffered system over RuCu/C catalyst. The results, shown in Fig. 12, indicated that the presence of PO_4_^3−^ can also effectively enhance the ammonia conversion and prevent the solution pH decreasing, similar to CH3COO^−^ ion.

### Reaction behavior between NH_4_^+^ and NO_2_^−^

It has been reported that NO_2_^−^ was an intermediate product which can be oxidized to NO_3_^−^ during the process of CWAO of ammonia at 200 °C. As shown in Fig. 10, the decreasing pH indicated that part of ammonia-A was oxidized to nitrite, however, the N_2_ selectivity is higher than 98% (shown in Fig. 6) and only very little nitrate was detected. Therefore, the effect of acetate anion on the behavior of the disproportionation reaction between NH_4_^+^ and NO_2_^−^ and nitrite oxidation reaction was investigated and the results are shown in Table 3. For comparison, the effect of pH was also included.

**Table 3:** Effect of pH and anions on the reaction between equimolar amounts of ammonia and nitrite.

Solution	pH	Ammonia left (ppm)	Nitrite left (ppm)	Nitrate Produced (ppm)	Ammonia conversion (%)
NH_4_Cl+NaNO_2_ (1)	10.5	448	451	0	10.4
NH_4_Cl+NaNO_2_ (2)	6.2	182	6	179	63.6
NH_4_Cl+NaNO_2_ (3)	3.6	252	0	245	49.4
CH_3_COONH_4_+NaNO_2_ (4)	6.2	169	110	70	66.2

Conditions: [NH_4_^+^]_0_ = 500 ppm, [NO_2_^−^]_0_ = 500 ppm, Air pressure = 2 MPa, reaction time = 3h.

As can be seen, pH strongly affected the two reactions. When pH was at 10.5, the reaction between NH_4_^+^ and NO_2_^−^ scarcely occurred, due to the very low percentage of NH_4_^+^ in alkaline environment and no detectable nitrate was formed. This implied that NO_2_^−^ can not be oxidized to NO_3_^−^, since the nitrite is predominantly present in the form of NO_2_^−^ at pH higher than 10. At pH 6.2, more than 63% of ammonia and most of the nitrite were converted. However, more than 30% of nitrite was oxidized to NO_3_^−^. With further decreasing pH to 3.6, the ammonia consumption reaction was restricted to some extent and more than 50% of ammonia was left due to the scarcity of NO_2_^−^, since HNO_2_ is becoming more dominant to NO_2_^−^ at a lower pH. In contrast with ammonia consumption reaction, the reaction of nitrite oxidation to NO_3_^−^ was further enhanced. The concentration of NO_3_^−^ reached a high value of 245 ppm, implying HNO_2_ was oxidized to NO_3_^−^.

Acetate anion exhibibt a strong influence on the nitrite oxidation reaction, although solution 2 and 4 (in Table 3) exhibited very comparable ammonia conversion. For solution 2, most of nitrite was converted, and some was reacted with NH_4_^+^ to form N_2_ and part of them was oxidized to nitrate. For solution 4, quite a lot of nitrite was left and only 70 ppm nitirate was dectected. This result indicated that part of nitrite in solution 4 neither reacted with ammonia (169 ppm was left) nor was oxidized to nitrate. The fact of little production of nitrate indicate that the nitrite in the solution 4 was maily present in the form of NO_2_^−^, not HNO_2_, although the pH was relatively acidic (6.2). One knows that the equilibrium constant of CH_3_COOH is much lower than that of HNO_2_. Therefore, it is reasonable to deduce that the equilibrium of CH_3_COO^−^/CH_3_COOH consumed H^+^ and made nitrite present in the form of NO_2_^−^. Another question is why quite an amount of ammonia was left and did not react with NO_2_^−^ to produce N_2_. This can be explained that the equilibrium of CH_3_COO^−^/CH_3_COOH made some ammonia present in the form of NH_3_, which can not react with NO_2_^−^. This was consistent with the fact the much higher reactivity of ammonia oxidation was attribute to the higher concentration of ammonia at acidic pH.

## Conclusion

The catalytic behavior for CWAO of ammonia to N_2_ was comparatively investigated in various ammonium salt solutions over Ru-Cu/C, Pt/TiO_2_ and Ru/TiO_2_ catalysts. It was found that the weak acid anion could increase the NH_3_ concentration in the solution and thus enhance the reactivity of catalytic oxidation of ammonia. The weak acid anion can also increase the concentration of NO_2_^−^ and facilitate the retro-disproportionation reaction between NH_4_^+^ and NO_2_^−^ toward N_2_. The both factors contribute the improving ammonia removal efficiency and/or the selectivity to N_2_.
